# Impact of computers in radiography: The advent of digital radiography, Part-2

**DOI:** 10.4103/0971-3026.41828

**Published:** 2008-08

**Authors:** BS Verma, IK Indrajit

**Affiliations:** Department of Radiodiagnosis and Imaging, Army Hospital (Research and Referral), Delhi Cantt. - 110 010, India

**Keywords:** Digital radiography, CR, DR, Flat panel detector, Tomosynthesis, Computer-aided diagnosis, Mobile DR

## Introduction

In the first part of this article, fundamental concepts regarding analog and digital signals, basic principal of radiography and simplified definitions of some frequently used terms in digital radiography were dwelt upon. Digital radiography systems were defined and categorized into Computed Radiography (CR) and Direct Digital radiography (DR) systems followed by a brief description of the CR systems.[1] Advantages and limitations of film screen radiography (FSR), digital radiography systems in general and CR systems in particular were also listed in a tabular form.

To recap, computed radiography (CR) systems use a photostimulable phosphor plate enclosed in a cassette. In CR, image acquisition is a two-stage process wherein image capture and image read out are done separately. Direct digital radiography (DR) systems, on the other hand, use detectors that have a combined image capture and image read out capability. DR systems are also called as DDR or ddR systems by some vendors. This second part of this article focuses on DR systems

### Direct digital radiographic systems

Cassettes form an important component in both film-screen radiography (FSR) and CR.[2] To improve workflow and to avoid the use of cassettes, a new class of detectors was manufactured that combined the processes of image capture and image read-out. This formed the evolutionary basis of DR systems. A functional overview of DR systems is given in Table 1.

**Table 1:** Advantages and limitations of direct digital radiography

Advantages	Limitations
• Increased workflow efficiency, saving time and labor	• High initial cost[3]
• Integrated high-powered x-ray system of 30-100 KW rating. Very short exposure time, eliminating motion blur	• Some radiographic views are difficult to obtain as the detectors are generally not free to be placed in any position
• Variable speed acquisition possible (speed class 100-800) depending on acceptable SNR. Reduction in radiation dose is possible as per the ALARA principle	• Careful handling is required due to fragile nature of most detectors
• Most DR systems have presets available for various anatomical studies including optimized post processing e.g. chest, spine etc.	
• Automatic tube detector positioning option for selected study	
• Auto selection of filter and focal spot size.	
• Automatic tracking for easy positioning	
• Automatic exposure control (AEC) facility	
• Immediate availability of image for quality check and diagnosis	

SNR: Signal to noise ratio; ALARA: As low as reasonably achievable; AEC: Automatic exposure control

There are four different types of DR Systems available depending on the type of detectors used in them[4,5] (a) Flat panel detector (FPD) based systems (b) 2D or ‘Area’ Charge coupled device (CCD) array based systems (c) Slot scanning type, and (d) Photon counting type.

### (a) Flat panel detector (FPD) based systems

**Figure 1:**
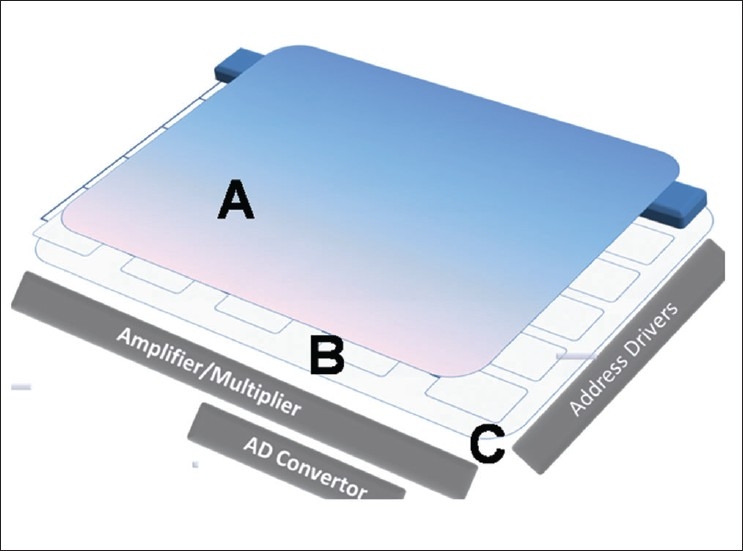
Flat panel detector assembly consisting of (A) an X-Ray converter, which is either a photoscintillator or a photoconductor; (B) TFT matrix and (C) glass substrate

FPD based DR systems are the most popular. In these, thin film transistor (TFT) arrays are used, which are made of amorphous silicon (a-Si). Silicon semiconductor sheets are etched with square detector elements, 70-200 µm on each side, on a glass substrate [Figure 1]. Each element has a capacitor and a switching transistor. Gait and drain lines are connected to each transistor and capacitor, enabling active readout of charges from each detector element separately. x-ray converter material is layered on the TFT matrix to make a flat panel detector.

There are two types of flat panel detectors depending on the method and material used for the conversion of x-rays into electrical signals[6,7]: (i) Direct X-ray conversion type that uses a photoconductor and (ii) Indirect X-ray conversion type with a photoscintillator/ phosphor screen. Key features of indirect and direct type of flat panel digital detectors are outlined in Table 2.

**Table 2:** Key features of indirect and direct type of flat panel digital detectors

Indirect digital conversion	Direct digital conversion
• Indirect conversion of X-rays to electrical signal	• Direct conversion of X-rays to electrical signal
• X-rays→ Light → Electrical signal	• X-rays→ Electrical signal
• Has a phosphor that converts X-rays to light and photodiode array that converts emitted light into electrical signals	• Uses a photoconductor that directly converts the absorbed X-rays to electrical signal without any intermediary light production
• Commonly used phosphors are Thallium doped Cesium Iodide or Gadolinium Oxy- Sulphide	• Detector material used is amorphous Selenium
• Light scatter reduces spatial resolution as well as noise due to aliasing	• No spread of signal as the applied high voltage immediately attracts and separates the electrons and holes produced by absorbed X-rays
• Generates poorer resolution images as the phosphor thickness is increased	• Maintains high resolution of images as the photoconductor thickness is increased
• Moderate fill factor depending on pixel size	• Perfect fill factor of nearly 100%
• High DQE for KV range used in conventional radiography	• Moderate DQE for conventional radiography but high DQE for mammography KV range
• Less sensitive to ambient temperature variations	• Very sensitive to ambient temperature variations

Direct conversion type of detectors use amorphous Selenium (a-Se) photoconductor. It is sandwiched between two electrodes to which high voltage is applied. When X-rays fall on this layer, electrons and holes are directly produced, in numbers proportional to the amount of X-rays absorbed. The applied high voltage immediately separates these electrons and holes, so that the signal does not spread. The electronic charge is stored in capacitors and is read out sequentially. Thus the X-rays are directly converted to electrical signal. These detectors have very high spatial resolution, moderate X-ray absorption efficiency (DQE) and an excellent fill factor.

Direct conversion technology has been largely derived from the experiences gained during the use of selenium drums in photocopier machines as well as in xeroradiography.[8] The relatively low atomic number of Selenium results in less X-ray absorption in general radiography KV range. The K-edge of selenium is more suited for the diagnostic KV range required in mammography.[9] As a result, direct conversion FPDs are more popular in mammography than in routine radiography.

**Figure 2:**
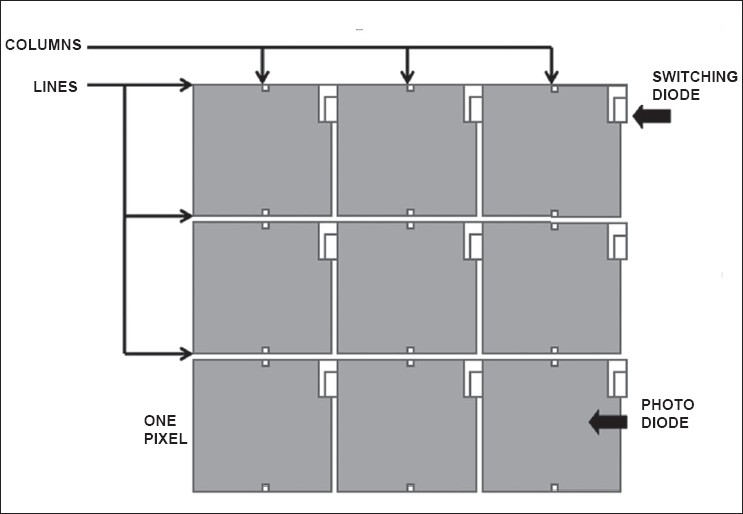
Schematic representation of a pixel layout in an Indirect type of FPD. An individual pixel is made of a single large area photodiode and a small area switching diode located at its corner. Switching diodes are connected to horizontal readout control lines, while photodiodes are connected to vertical data columns

Indirect conversion FPDs first convert x-rays to light in a scintillator/phosphor, which in turn is then detected by photodiodes and TFT arrays [Figure 2]. Thallium-doped Cesium Iodide (CsI) is the most commonly used phosphor material. It is structured in the form of thin needle-shaped crystals. The light produced by X-rays in these crystals is channeled by internal reflection and does not spread, thereby maintaining a good spatial resolution. Another material used is turbid Gadolinium Oxy- Sulphide or Gadox (Gd_2_O_2_S). It has an amorphous structure that permits spread of light produced by X-ray absorption, resulting in poorer spatial resolution. CsI-based detectors are more efficient in x-ray absorption than Gadox detectors and have a better DQE, but they are fragile and the detectors need to be handled with the utmost care. Most flat panel detectors are available in large sizes e.g. 43×43 cm, 41×41 cm, 43×35 cm etc.[10]

## Other Types of DR Systems

### (b) 2D or ‘Area’ CCD array-based systems

In these DR systems the x-rays are absorbed and converted into visible light in large scintillators or phosphors.[11] This light is channeled by means of mirrors/lenses/prisms and fiberoptically coupled to a much smaller light-sensitive CCD array. As the CCD arrays are small (2-5 cm) in size and have very small (10-20 µm) detector elements, demagnification is required. These detectors are relatively bulky, but are less costly than FPDs. One such system, Xplorer from Image Dynamics, has a pixel size of 108 µm and an image area of 43 × 43 cm. Some vendors have introduced Complementary Metal Oxide Semiconductor (CMOS) based systems in place of CCD. The salient features of CCD based DR systems are outlined in Table 3.

**Table 3:** Advantages and limitations of charge-coupled device (CCD) based DR systems

Advantages	Limitations
• Relatively cheaper	• Bulky design
• Individual defective components can be replaced rather than changing the entire detector	• Relatively small CCD arrays (2-5 cm) than the typical projected X-ray areas require image demagnification and optical coupling[7]
• Upgradeable with future innovations and advancements in technology	• Optical system noise degrades image quality
	• Lens system introduces geometric distortions and optical scatter reducing spatial resolution
	• Defects in the fiber optics may cause structural artifacts in the acquired image
	• Thermal noise in the CCD can degrade image quality. This is mitigated with the use of cooled CCDs by some vendors
	• Repeated exposure to X-rays may damage the optical system and the electronics in the long run. Some manufacturers design systems to protect electronics from X-rays

### (c) Slot-scanning types

These systems use a narrow fan beam that moves across the anatomical region. Two precisely aligned moving slit collimators, one on either side of the patient, are used in such systems. This prevents scatter radiation from reaching the detector. Thus use of a radiographic grid is not necessary, significantly reducing the radiation dose. These systems use a narrow CCD array with few rows of detectors to scan the patient anatomy. A technique called time delay integration (TDI) is used to transfer information from one row of detectors in the CCD to the next row as the gantry passes over a body part. Thus the image information about a given section of body gets reinforced, effectively increasing the SNR. It works somewhat like a scanogram in CT though here the gantry moves while the patient remains stationary. Long body anatomy can be covered in a continuous manner by these systems. One such system (Statscan by Lodox) can cover the entire body in 13 seconds.[12] These units can take different views without moving the patient. Long exposure times require high capacity X-ray generators and tubes. Salient features of slot scanning type of DR systems are outlined in Table 4.

**Table 4:** Advantages and limitations of slot-scanning DR systems

Advantages	Limitations
• Radiation dose is reduced as grid is not required	• High initial cost
• Longer and larger anatomical regions are well covered	• Bulky design
• Useful in radiography of trauma patients, orthopedics, emergency[13] and disaster care	• Exposure time is long
	• Requirement of equipment with high rating of generators and X-ray tubes
	• Patient motion may degrade image quality

### (d) Photon counting type DR system

Photon counting type of DR system has construction similar to the slot scanning type described above but uses a different type of detector. These systems use a multislit detector made of crystalline Silicon (Si) as a scintillator. Principle is similar to the one used in direct type of flat panel detectors. A voltage of about 100 volts is applied across the array of thin (50 µm) Si crystals. Absorbed X-rays produce electrons and holes. Each of these events is counted in a meter with time corresponding to the spatial location along the direction of X-ray fan beam sweep. Each absorbed X-ray photon results in a unit count regardless of the photon energy. As the electrical pulse generated is much higher than the electronic noise, this type of DR systems produce images with high SNR. Currently this technology is being used for mammography (Sectra microdose). Another DR system requiring low radiation dose (EOS of Biospace med) uses gaseous microstrip detectors for biplane whole body imaging in erect weight bearing position, with excellent results. Salient features of Photon counting type DR system are outlined in Table 5.

**Table 5:** Advantages and limitations of photon counting type DR systems

Advantages	Limitations
• Radiation dose is reduced as grid is not required	• Exposure time is long
• High system DQE	• Requirement of equipment with high rating of generators and X-ray tubes
• High SNR due to minimal electronic noise	• Patient motion may degrade image quality
• No ghost image (previous exposure residue)	
• High contrast and detail resolution	

## Available Configurations of DR Systems

**Figure 3:**
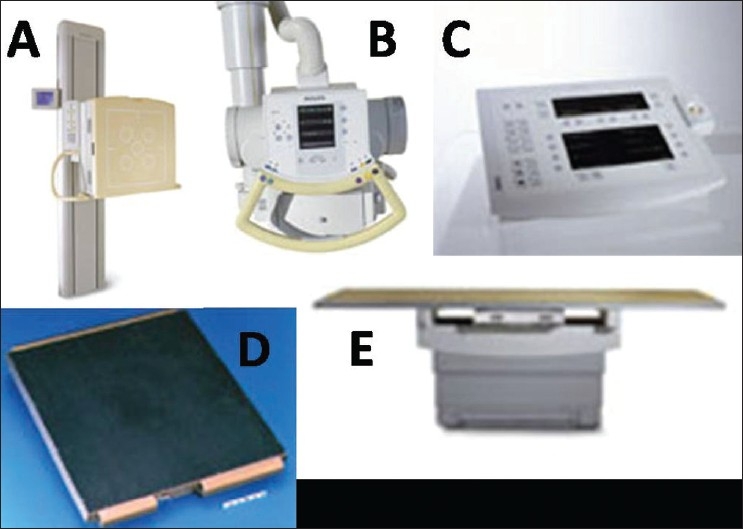
Diagram depicting various parts of a direct digital radiography system (A) vertical stand. (B) tube. (C) console. (D) detector. (E) couch

Most of the DR systems have an integrated X-ray tube assembly that may have any one of the following configurations: Ceiling mounted, floor mounted or mounted on an arm with the digital detector assembly mounted on the second arm.[11] Two detectors configuration usually has a wall/floor mounted stand for erect radiography and a height adjustable table with floating table top for recumbent radiography. Single, tiltable, wall/floor mounted detector along with a ceiling mounted tube and a mobile patient trolley may be able to do most erect and recumbent studies with a single detector. Similarly, X-ray tube and detector mounted on a U arm, combined with a mobile patient trolley, can perform most examinations. The parts of a typical DR system is outlined in Figure 3.

## FPD Attributes

### (a) Field of View:

The detector size offered by various vendors is under constant technological modifications. At present the maximum detector size commonly available is 43×43 cm (17×17 in) in a four pieces tiled configuration. Such a detector made by Trixell is used in DR systems of Philips, Siemens and Carestream/ Kodak. The DR systems manufactured by GE Medical, currently has a 41×41cm single piece detector. Canon makes detectors in various sizes from 43×43 cm to 28.8×22.6 cm (11×9 in). The commonest sizes available currently are 43×43 cm and 35×43 cm.

### (b) Pixel size:

All digital detectors are made of arrays of pixels (strictly speaking these should be called detector elements or dels). Spatial resolution in digital imaging is a function of pixel size, with a smaller pixel yielding higher resolution. A variety of pixel sizes of detectors are offered by different vendors. Trixell offers detectors with a pixel size of 143 µm, while Canon produces 160µm for larger detectors and 100 µm for smaller (11×9 in) one. GE detector pixel is 200 µm.

### (d) Type of detectors:

Trixell, GE and Canon detectors are all indirect type. Hologic used a direct type of detector in their DR system Directray.

## Innovations and Newer Applications in DR

Digital Radiography is witnessing rapid innovations in hardware as well as its software applications. Few of the exciting applications are mentioned below. Clinical utility and the true potential of these applications will be understood better in the years ahead.

### (a) Tomosynthesis:

In this technique multiple low dose exposures are given from various angles while the X-ray tube moves in an arc and the detector remains stationary. Multiple images with different focal zones are possible to be created by addition of these low dose images after pixel shift. It emphasizes contrast in a particular layer of a region of body.[14] Generated images can be viewed singly or as a cine loop. It is considered useful in Chest, IVU studies and mammography.[15]

### (b) Dual-energy imaging:

By using a high and low kilo-voltage technique, two datasets are created. Soft tissues and bones can be separately depicted by this method.[16] Dual-energy techniques are most effective when both images are acquired simultaneously.[14] Similar results are obtained with two exposures within a very short period of time. This is useful in chest radiography, particularly for the evaluation of partially calcified nodules and pleural plaques.[14]

### (c) Computer aided diagnosis (CAD) software programs:

These are important in early detection of cancer of the lung and breast. The suspicious areas are marked by the software for review by the radiologist. The efficiency of CAD software program is related to its sensitivity and specificity profile. These programs are gradually improving with newer generations/editions having better sensitivity and specificity profile. The main advantage of CAD is that it permits a radiologist to avoid overlooking diagnostically significant findings.

### (d) Automatic image stitching:

This is a feature that is useful in determining precise measurements in lengthy anatomical regions like the spine or lower limbs. The largest flat-panel DR plates available today are 43 × 43 cm. Using these detectors, only a limited portion of the body part can be imaged at one given time, thus making these detectors inadequate for studying the whole spine or the entire lower limb. To overcome this problem, multiple sequential exposures at different patient positions are acquired in a still patient. Automatic stitching is then performed to reconstruct a larger composite image. This special software enables pixel shift and overlap.

### (e) Mobile DR:

This is in general a 17 × 14-in flat panel detector connected by a cable to a mobile x-ray system having a monitor. The use of mobile DR systems is hampered by the fragility of the FPDs and the high costs. A mobile DR system, when compared with an FSR system, avoids problems related to the availability, storage, transportation and disposal of films and chemicals.[17]

### (f) Wireless FPDs:

With the introduction of the model Pixium 3543 from Thales, wireless portable DR is now a reality. It wirelessly transfers image data to the DR system. Alternatively the image data can be transferred to DR console via an Ethernet cable. It has no cables and does not interfere with surrounding machines. Typically a 17 × 14-in image is made available within 3 s.[18] This allows radiography of difficult regions of the body like the axilla or the TM joint and enables radiography in unusual positions as in a flexed knee, or in a limb with limited mobility due to contractures, etc.

### (g) Fluoroscopy and radiography:

Real-time digital imaging in DR is possible with the Pixium RF 4343, from Thales. It facilitates high-quality radiography and fluoroscopy (up to 30 images/s).[18] The fluoroscopy feature is of use in gastroenterology, urology, and vascular applications. Newer FPDs like Pixium 4700 and 4800[18] from Thales are used for vascular and cardiovascular DSA applications by permitting low-dose fluoroscopy.

## Newer Innovations and Applications in CR

Some of the drawbacks of CR systems, namely cassette handling, long read out time of PSP plates, low DQE and poor resolution have been addressed by newer innovations and technological advances.

### (a) Automated CR systems with fast readout:

CR systems efficiency has been recently improved by reducing the read out time and by removing the step of cassette handling. Automated CR systems achieve this by line-scan lasers and photodiode detectors that reduce the readout time of a PSP plate to less than 10 s.[19] In these systems there is no cassette handling, leading to totally automatic image data acquisition.

### (b) Newer phosphors for PSP plates:

Commercially available PSP plates have unstructured phosphor like rubidium chloride or barium fluorohalides doped with Europium. These are scanned in a raster pattern. A needle-shaped phosphor cesium bromide, has been newly introduced, for example, in Konica Minolta's Regius 370 Upright DR, and is considered more efficient due its structured configuration of crystals.[20] It reduces light diffusion because of the needle shaped configuration that acts as light guide. In addition the newer phosphors are more efficient with an increased DQE.

### (c) Mobile CR systems:

Bedside radiography of critically ill patients with conventional CR involves physical transport of the cassette to the CR reader, often located far away. The situation gets worse as the number of ‘portable’ films increase. To save labour, time and improve workflow, portable compact CR systems have been introduced in late 2007, with Fujifilm Go (FCR Carbon XL CR reader) and Carestream Health Inc (Pointof-Care CR-ITX 560) machines. These systems basically have a mobile X-ray unit with an integrated CR reader. They are easy to use and offer quick image availability in less than 25 s.

## Impact of DR on Departmental Workflow

Plain radiography accounts for 50 to 70% of the total workload in a large radiology department even today.[21] Study of the workflow in radiography is therefore important in daily practice. Let us briefly examine the issues that govern workflow in radiography.

The process of FSR workflow ranges from patient registration to the availability of dried radiographic film. Analysis of these individual steps may help in finding ways to increase the workflow. The process consists of the following key steps: (a) entering patient information into the register/console, (b) setting exposure parameters, (c) getting and positioning the radiographic cassette, (d) positioning the patient, (e) radiographic exposure, (f) film processing, (g) cassette reloading, and (h) image quality check before the patient goes away.[22]

It is evident from above that a technological move from FSR to CR does not eliminate or reduce the duration of any of the described steps. As a result, workflow does not significantly improve by introducing digital radiography in the form of CR. However, in DR, the steps (c), (f), and (g) are totally eliminated, significantly improving the workflow. Availability of HIS/ RIS to automatically populate the patient information in equipment consoles enhances the workflow significantly in both CR and DR systems. 

## Conclusion

Conventional Radiography is evidently the last of the radiology modalities to embrace and incorporate digital technology. By their tremendous impact on the image quality and the workflow, digital radiography systems have become practicable alternatives.[23] CR is a simple and cost effective technology that permits use of existing radiographic equipment. It has been suggested that for moderate workload (upto 50-60 films per day), a CR system is adequate. High cost of a DR system is justified only when the workload is much beyond this level. 

The current scenario in CR and DR is one of relentless technological advancement and expansion. CR systems now have features that traditionally had been associated with DR. Similarly conventional X-ray machines can now be equipped with a DR detector as a retrofit, saving greatly in costs as compared to purchase of a new DR systems. As a fallout of these developments, the distinction between the CR and DR technologies is blurred.[19]

Lastly, a change over to digital technology is essential to create a fully digital ‘filmless’ radiology department and fully reap the benefits of implementing RIS and PACS programs.

## References

[1] Verma BS, Indrajit IK (2008). Advent of digital radiography: Part 1. Indian J Radiol Imaging.

[2] Seibert JA Overview of Digital Detector Technology, University of California, Davis. http://www.aapm.org/meetings/05AM/pdf/18-2623-22086-53.pdf.

[3] Aarati A (2007). Is CR dead? Imaging Management. http://www.imagingmanagement.org.

[4] Padovani R Basic Principle of Flat Panel Imaging Detectors 2006. http://www.dimond3.org/Trier_2006/Basic%20principles%20of%20flat%20panel.pdf.

[5] The challenges of direct digital X-ray detectors. A review of digital detectors in medical X-ray technology. http://www.dondickson.co.uk/download/Challenges_of_Direct_Digital_Radiology.pdf.

[6] Chabbal J, Arques M, Peyret O Flat panel X-ray detector. http://beaune.in2p3.fr/sessions/chabbal.pdf.

[7] Chotas H, Dobbins J, Ravin C (1999). Principles of digital radiography with large area electronically readable detectors: A review of basics. Radiology.

[8] Paulus DD (1980). Xeroradiography: An in-depth review. Crit Rev Diagn Imaging.

[9] Smith AP Fundamentals of digital mammography. Physics, technology and practical considerations. http://www.hologic.com/oem/pdf/R-BI-016_Fund-Dig%20Mammo.pdf.

[10] Bruijns T, Stueve D Image quality assessment in digital X-ray detection systems Philips medical systems. http://www.aapm.org/meetings/04SS/documents/bruijns.PDF.

[11] Yester M An overview of digital imaging systems for radiography and fluoroscopy, Ph.D. University of Alabama at Birmingham.

[12] Lodox Systems http://www.lodox.com/html/product.html.

[13] Beningfield S, Potgieter H, Nicol A, van As S, Bowie G, Hering E (2003). Report on a new type of trauma full-body digital X-ray machine. Emerg Radiol.

[14] Flynn M Technical advances in CR and DR Part 2. http://www.imagingeconomics.com/issues/articles/2004-04_16.asp.

[15] Poplack SP, Tosteson TD, Kogel CA, Nagy HM (2007). Digital breast tomosynthesis: Initial experience in 98 women with abnormal digital screening mammography. AJR Am J Roentgenol.

[16] Smith R, CR, DR Today and Tomorrow Dec 2004. http://www.imagingeconomics.com/issues/articles/2004-12_05.asp.

[17] Cho KH, Freckleton MW (2002). Digital radiography for the field: A portable prototype. J Digit Imaging.

[18] Pixium Product documentation. http://www.trixell.com/html-gb/products/interne/product_documentation.html.

[19] Seibert JA Digital radiography: CR versus DR? Time to reconsider the options, definitions, and current capabilities. http://www.fujimed.com/products-services/imaging-systems/digital-xray/doc/AR_12-07_Fuji_Seibert.pdf.

[20] Konica Minolta.s CR systems shine at RSNA. http://www.healthimaging.com/content/view/9120/89/.

[21] Romlein J CR versus DR: Blurred lines of distinction. http://www.fujimed.com/products-services/imaging-systems/digital-xray/doc/AR_12-07_Fuji_Romlein.pdf.

[22] Wideman C, Gallet J (2006). Analog to digital workflow improvement: A quantitative study. J Digit Imaging.

[23] Andriole KP, Luth DP, Gould RG (2002). Workflow assessment of digital versus computed radiography and screen-film in the outpatient environment. J Digit Imaging.

